# Construction Notes

**Published:** 1897-01-30

**Authors:** 


### CONSTRUCTION NOTES.

### New Laundry Offices, Edinburgh Royal Infirmary.

These buildings have been so planned that the patients' laundry and the staff laundry are separate from each other, though under the same roof. The drying rooms are in two storeys. This is the arrangement adopted in almost all modern buildings of this kind. It gives excellent results, and effects a great economy in space. Lifts and staircases connect the two floors. The lower floor is provided with sliding drying horses, while the upper one has fixed bars. On the first floor, and in a small tower-like block, at the south-west angle, sleeping accommodation is provided for thirty-one laundrymaids in separate rooms or cubicles. Besides this is a large dining-room with windows to the south and west, kitchen, lavatories, baths, &c.; a separate sitting-room and bath-room are provided for the chief laundrymaid. The buildings are designed to be of stone with slate roofs, and to be, as far as possible, fireproof. The machinery for washing, drying, and ironing is to be of the most modern and approved type. The architects are Messrs. Sydney Mitchell and Wilson, of Young Street, Edinburgh.

